# Electrochemical Corrosion Behavior and the Related Mechanism of Ti_3_SiC_2_/Cu Composites in a Strong Acid Environment

**DOI:** 10.3390/ma17164035

**Published:** 2024-08-14

**Authors:** Rui Zhang, Chengzhi Du, Fuyan Liu, Chenlong Wu

**Affiliations:** 1 School of Mechanical Engineering, Chengdu University, Chengdu 610106, China; duchengzhi@stu.cdu.edu.cn; 2 State Key Laboratory of Solid Lubrication, Lanzhou Institute of Chemical Physics, Chinese Academy of Sciences, Lanzhou 730000, China; 3 School of Chemical Engineering and Materials, Changzhou Institute of Technology, Changzhou 213032, China; 4 School of Mechanical Engineering, Xinjiang University, Urumqi 830000, China

**Keywords:** Ti_3_SiC_2_/Cu, passivation behavior, electrochemical corrosion, corrosion mechanism

## Abstract

The electrochemical corrosion behaviors of Ti_3_SiC_2_/Cu composites in harsh media including dilute HNO_3_ and concentrated H_2_SO_4_ were studied in detail and the related corrosion mechanisms were explored. Under open-circuit potential, the corrosion resistance of Ti_3_SiC_2_/Cu in dilute HNO_3_ was worse than that in concentrated H_2_SO_4_. In dilute HNO_3_, Ti_3_SiC_2_/Cu exhibited a typical passivation character with a narrow passivation interval. During the corrosion process, the dissolution of Cu-Si compounds resulted in the destruction of the passivation layer on the surface. Additionally, with the increasing of the potentials, the oxidation of Cu and Si atoms led to the generation of the oxide film again on the surface. In concentrated H_2_SO_4_, the Ti_3_SiC_2_/Cu composite was covered by a double-layered passivation layer, which was composed of an internal layer of TiO_2_ and an external layer of Cu_2_O and SiO_2_. This was because Cu diffused into the surface and was oxidized into Cu_2_O, which formed a denser oxidized film with SiO_2_. In addition, it was found that Ti_3_SiC_2_/Cu has better corrosion resistance in concentrated H_2_SO_4_.

## 1. Introduction

Since the preparation of the MAX phase, research on its properties and applications, such as electrical conductivity, thermal conductivity, machinability, bending resistance, superconductivity, and optical property, has continuously emerged [1,2,3]. As an important character of ceramics, the corrosion resistances, including frictional corrosion, hydrothermal corrosion, resistance to strong acid and alkali corrosion, electrochemical corrosion, etc., have been substantially studied [4,5]. It is well known that electrochemical corrosion generally occurs in some circumstances, such as in the atmosphere, sea, acid or alkali solutions, and wastewater. Currently, metals or alloys are used as the structural parts. If corrosion happened, especially under the influence of mechanical action, the property of materials was impaired, and even some catastrophes happened [6,7]. The corrosive behaviors of Ti_3_SiC_2_ in various acid, alkali and salt solutions have been studied, and it was found that the formation of oxides in its service environment was beneficial for the improvement of its corrosion resistance. However, the effect of the passivation film on the electrochemical corrosion of Ti_3_SiC_2_ has been less studied [6,8]. Up until now, the corrosive behavior of Ti_3_SiC_2_ in HCl and H_2_SO_4_ has been thoroughly investigated. In both acids, under the open potential and high anode potential, Ti atoms were leached out and Si atoms were oxidized in situ, forming a passivation film [9]. Barsoum et al. [10] studied the electrochemical behaviors of some common MAX phases in strong acids and alkali solutions (such as HCl, H_2_SO_4_, and NaOH) and they found that the corrosion resistance of Ti_3_SiC_2_ was better than that of pure Ti, which resulted from the formation of the thin passivation film of SiO_2_. Ming Zhu et al. [9] investigated the electrochemical behaviors of Ti_3_SiC_2_ in the 3.5% NaCl solution, and they discovered that it exhibited excellent corrosion resistance. However, the passivation behavior of Ti_3_SiC_2_ was weaker than Ti, because the special layered structure of Ti_3_SiC_2_ provided a path for the spread of Si, which resulted in the low passivation efficiency of atoms diffusing to the obstacle layer.

Previously, we have investigated the static corrosion of Ti_3_SiC_2_/Cu composites in a strong acid environment and the electrochemical corrosion in a 3.5% NaCl environment, explaining the corrosion mechanisms involved [11,12]. However, in industrial production environments, most of the structural components are exposed to acidic conditions [13]. In this study, the electrochemical corrosion resistance of Ti_3_SiC_2_/Cu composites in the harsh media of dilute HNO_3_ and concentrated H_2_SO_4_ has been thoroughly investigated in the context of strong-acid and electrochemical corrosion environments, and the related corrosion mechanisms have been discussed.

## 2. Experimental

### 2.1. Sample Preparation

Ti_3_SiC_2_/Cu composites were synthesized by the spark plasma sintering (SPS) process. The original Ti_3_SiC_2_/Cu powder mixtures were composed of 15 vol.% Cu powder (average grain size: 74 μm, purity ≥ 99.9%, China Shanghai McLean Biochemical Co., Ltd., Shanghai, China) and 85 vol.% Ti_3_SiC_2_ powder (average grain size: 38 μm, purity ≥ 98%, China Jilin 11 Technology Co., Ltd., Jilin, China). The powder mixtures were uniformly mixed, then sintered by an SPS furnace (SPS, Model Labox-350, SINTER LAND INC., Niigata, Japan) and finally cooled with the furnace. The sintering temperature was 1000 °C. The sintering pressure was 35 MPa. The swelling duration was 20 min. This Ti_3_SiC_2_/Cu preparation scheme has been reported previously, and it is confirmed that it has high mechanical properties such as hardness and compressive strength. Not only that, its excellent tribological properties have been widely studied [14,15]. The diameter of the as-prepared composite was 25 mm and its thickness was 10 mm. The relative density of the composites was 95%~96%. The as-synthesized composites were processed into pieces (10 mm × 10 mm × 5 mm) by wire cutting. These pieces were polished by diamond grinding discs to 3000 mesh. Then, they were ultrasonically washed in acetone and ethanol in turn, followed by drying in air at room temperature. Finally, the pieces were linked by Cu wires and sealed with epoxy to prepare a work electrode.

### 2.2. Electrochemical Measurements

Electrochemical measurements were conducted on a DH7003 potentiostat/galvanostat controlled by DHMultiElec software (V6.21.9.23h) linked by a general three-electrode electrochemical cell full of dilute HNO_3_ (11.6 wt%) and concentrated H_2_SO_4_(98 wt%) solutions, at room temperature. The counter electrode was the Pt electrode and the reference electrode was the Ag/AgCl electrode (0.197 V vs. standard H electrode, SHE). These three electrodes were correctly positioned in the fitted locations of the cell. Potentiodynamic polarization experiments were performed from −0.3 to 3 V (vs. open-circuit potential (OCP)). The scan rate was 0.333 mV/s. It has been stated in the relevant literature that the potential scan rate plays an important role in minimizing the effects of aberrations in Tafel slope and corrosion current density analysis, and that the 0.333 mV/s used has no deleterious effect on the Tafel extrapolation method for determining the corrosion current density of test samples [16,17,18]. The electrochemical impendence spectroscopy (EIS) data gained by immersion in dilute HNO_3_ (11.6 wt%) and concentrated H_2_SO_4_ (98 wt%) solutions for 30 min at the open potential, or by potentiostatic polarization at different potentials for 12 h, were acquired by changing the frequency from 100 kHz to 10 mHz, with the current amplitude of 5 mV.

### 2.3. Characterization

The surface morphology and composition of the composites before and after immersion in harsh media were characterized by scanning electron microscope (SEM, JIB-4700F, JEOL, Akishima City, Tokyo, Japan) affiliated by an energy-dispersive spectrometer (EDS). The chemical valence states of the passivation layers generated during potentiostatic polarization were examined by X-ray photoelectron spectroscopy (XPS, PHI 5000 VersoProbe III, Ulvac-PHI, Akishima City, Tokyo, Japan). The X-ray beam spot was <10 μm. The accelerating voltage was 5 kV. Its energy resolution was <0.5 eV and its vacuum pressure was 5 × 10^−8^ Pa. The C 1s with a binding energy of 284.8 eV was used as a reference for all chemical elements (including Ti, Si, C, O, and Cu).

## 3. Results and Discussion

### 3.1. Potentiodynamic Polarization

**Figure 1:**
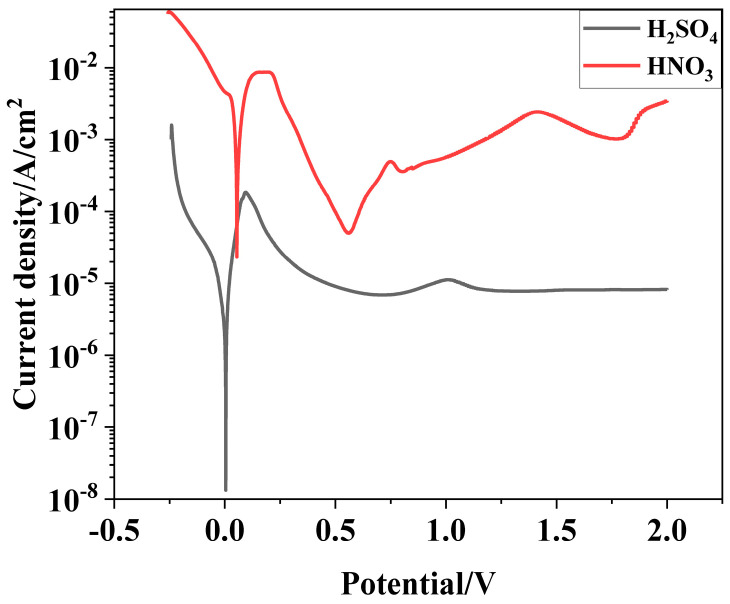
Polarization curves of Ti_3_SiC_2_/Cu composites in dilute HNO_3_ (11.6%) and concentrated H_2_SO_4_.

Potentiodynamic polarization curves of Ti_3_SiC_2_/Cu composites in dilute HNO_3_ and concentrated H_2_SO_4_ solutions are shown in Figure 1. A passivation behavior was observed for the composites under anodic polarization conditions, but the passivation gap of the composites in the dilute HNO_3_ was very narrow, which resulted from the weak stability of the as-formed oxide film of the composites. In dilute HNO_3_ (11.6%), the anodic curve of the Ti_3_SiC_2_/Cu composites exhibited a tipping point at 0.2 V, and subsequently underwent a passivation region, with a passivation current density of 5.01 × 10^−5^ A/cm^2^. When the voltage was higher than 0.55 V, the anode current density of Ti_3_SiC_2_/Cu obviously showed a quick upward tendency, which resulted from the destruction of the passivation layer, resulting in the accelerating of corrosion. When the voltage was in a range of 1.4 V–1.8 V, the anode current density of Ti_3_SiC_2_/Cu decreased. The reason was that Ti atoms were oxidized to TiO_2_, forming an oxide film. When the potential was greater than 1.8 V, the anodic current density showed a step-up trend, which may be due to hydrogen precipitation corrosion on the material surface. In the concentrated H_2_SO_4_ solution, the anodic curve of the Ti_3_SiC_2_/Cu composites exhibited a tipping point at 0.1 V, and it subsequently underwent a relatively wide passivation region, with a passivation current density of 6.87 × 10^−6^ A/cm^2^. This was because a double-layered passivation layer made up of TiO_2_ and SiO_2_ was formed on the surface of the composites. While the voltage was in the range of 1 V–2 V, the current density of Ti_3_SiC_2_/Cu slightly decreased and remained around 8.09 × 10^−6^ A/cm^2^, which resulted from the transformation of Ti from oxides (II) to TiO_2_.

As seen in Table 1, the open potentials of Ti_3_SiC_2_/Cu composites in two strong acids were similar. However, the self-corrosion current density of the composites in dilute HNO_3_ and concentrated H_2_SO_4_ were 7.05 × 10^-4^ A/cm^2^ and 3.01 × 10^-6^ A/cm^2^, respectively. Therefore, at the open potential, the corrosion resistance of the composites in concentrated H_2_SO_4_ was superior than that in dilute HNO_3_. When the potential was higher than 1 V, the Ti_3_SiC_2_/Cu composites exhibited a secondary passivation phenomenon in two strong acids.

### 3.2. EIS

**Figure 2:**
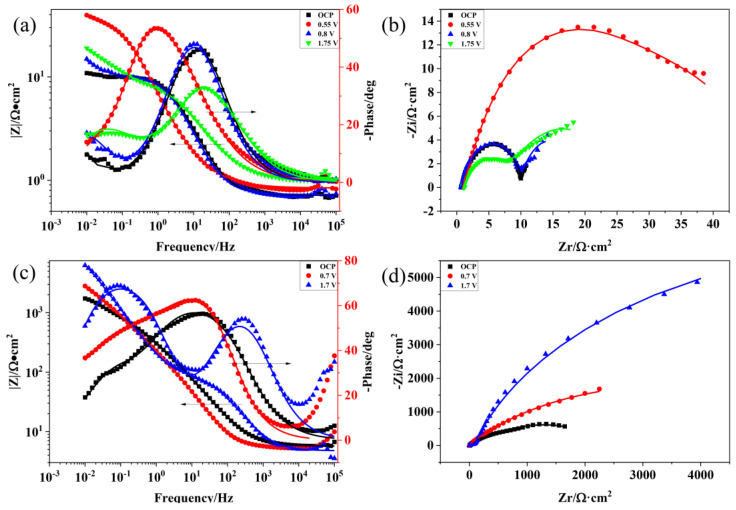
Bode and Nyquist plots of Ti_3_SiC_2_/Cu composites soaking in dilute HNO_3_ (11.6%) and concentrated H_2_SO_4_ for 30 min under OCP and potentiostatic polarization under different potentials for 12 h: (**a**) Bode plot of Ti_3_SiC_2_/Cu in dilute HNO_3_ (11.6%), (**b**) Nyquist plot of Ti_3_SiC_2_/Cu in dilute HNO_3_ (11.6%), (**c**) Bode plot of Ti_3_SiC_2_/Cu in concentrated H_2_SO_4_ and (**d**) Nyquist plot of Ti_3_SiC_2_/Cu in concentrated H_2_SO_4_.

For further investigating the corrosion process of the Ti_3_SiC_2_/Cu composites, the EIS data of the composites in two strong acids were acquired at the open potential and different polarization potentials (see Figure 2). As shown in Figure 2a,c, at high frequencies, the value of log|Z| tended to be stable and the phase angle was near zero, which indicated that the impedance was dominated by the solution resistance (R_s_). As shown in Figure 2a, when the polarization potential increased, the phase curve of the composites moved to left first, and subsequently moved to right. It was speculated that the passivation layer first became thin, and then became thick [19]. At the passivation interval, the oxide film of the composites was relatively thin. There was a platform at the high frequency of the Bode diagram, which related to the solution resistance (R_s_). Under the medium and low frequencies, the slope of log|Z|~logf tended to be −1 and the phase angle approached −90°. At high frequencies, a platform was seen in the Bode diagram, which was related to the solution resistance (R_s_). As the voltage increased, the capacitive arc radius of the Ti_3_SiC_2_/Cu composites first rose, then declined (see Figure 2b). At 0.55 V, the corrosion resistance of the composites was the best. At the open potential (OCP) and 0.7 V, the capacitive arc radius was similar, which indicated that the composites showed a similar corrosion resistance behavior at OCP and 0.7 V. 

In concentrated H_2_SO_4_, the Bode plots tend to increase at higher frequencies when the potentials are 0.7 V and 1.7 V (see Figure 2c). As seen in Figure 2d, with the increase in the potential, the capacitive arc radius of the Ti_3_SiC_2_/Cu composites in the concentrated H_2_SO_4_ increased and the corrosion resistance of the composites was the best at 1.75 V. The capacitive arc radius of the Ti_3_SiC_2_/Cu composites in dilute HNO_3_ was much smaller than that in concentrated H_2_SO_4_, which suggested that the corrosion resistance of the composite in concentrated H_2_SO_4_ was better. The impedance characteristics and corrosive behaviors of the composites in different situations were represented by the appropriate equivalent circuit. Based on the point-defect model (PDM), a passivation layer had a double-layered structure. In accordance with the PDM, the passivation process can be represented by the two chemical reactions below, which interpret the growth of the film and the transformation of the matrix, respectively [20].

(1) $$M+\frac{x}{2}H_{2}O=[M_{M}+\frac{x}{2}O_{O}]+x\delta^{-}$$

M → M^δ+^ (aq) + V_m_ + δe^−^ (2)

**Figure 3:**
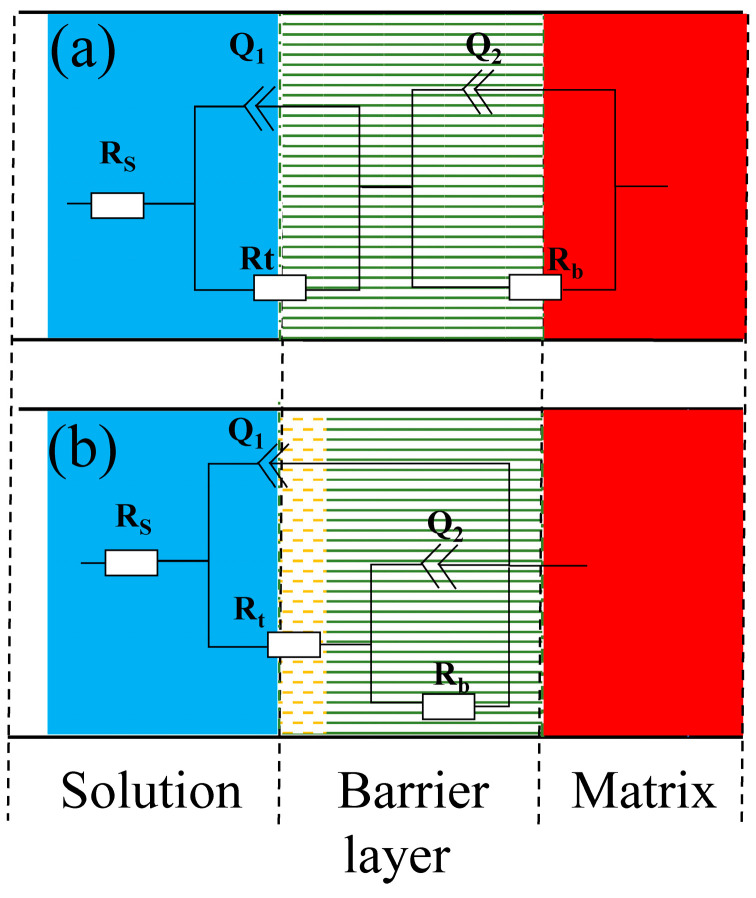
Equivalent circuit diagrams of Ti_3_SiC_2_/Cu composites in dilute HNO_3_ (11.6%) and concentrated H_2_SO_4_ at different potentials: (**a**) Ti_3_SiC_2_/Cu in dilute HNO_3_ (11.6%) at different potentials (such as OCP, 0.55 V, 1.75 V) and in concentrated H_2_SO_4_ at 1.7 V; (**b**) Ti_3_SiC_2_/Cu in dilute HNO_3_ (11.6%) at 0.8 V and in concentrated H_2_SO_4_ at OCP and 0.7 V. (R_s_ represents the solution resistance. Q_1_ and R_t_ represent the electric double-layer capacitance and charge-transfer resistance, respectively. Q_2_ and R_b_ represent barrier capacitance and resistance, respectively.)

Two time-constants were determined by combining PDM and the feature of impedance pattern, then a suitable equivalent circuit was applied to fit the experimental data [12,16,21]. As seen in Figure 3, the EIS data of the Ti_3_SiC_2_/Cu composites in dilute HNO_3_ at OCP, 0.55 V and 1.75 V, and in concentrated H_2_SO_4_ at OCP and 1.7 V were fitted by the equivalent circuit of R_s_(Q_1_R_t_)(Q_2_R_b_). Meantime, the EIS data of the Ti_3_SiC_2_/Cu composites in dilute HNO_3_ at 0.8 V and in concentrated H_2_SO_4_ at 0.7 V were fitted by the equivalent circuit of R_s_((Q_1_R_t_(Q_2_R_b_))). In dilute HNO_3_, when the potential was 0.55 V, the generation of a single-layer oxide film benefitted the corrosion resistance of the composites. When the potential was 0.8 V, a porous double-layer oxide film was formed, and its inner oxide film showed relatively poor corrosion resistance. At 1.75 V, the corrosive current density of the composites substantially decreased, because at low overpotentials the oxide film of the Ti-containing materials transformed from the noncrystalline state to the crystalline state, forming a densified oxide layer on the surface of the composites. In concentrated H_2_SO_4_, when the potential was 0.7 V, a double-layer oxide film was formed on the surface of the composites, which was favorable for its corrosion resistance. When the potential was 1.7 V, a single-layer oxide film was formed, and its corrosion resistance was further enhanced.

Polarization resistance (R_p_) can be thought of as the association of R_b_ and R_t_. According to the fitted results (see Table 2), the corrosion resistance of the Ti_3_SiC_2_/Cu composites in concentrated H_2_SO_4_ was superior. At 0.55 V, the R_b_ value of the Ti_3_SiC_2_/Cu composites in dilute HNO_3_ was relatively high, which was due to the generation of a single-layer oxide film on its surface. As the potential increased, for example, at 0.7 V, the oxide film of the composites converted from the single-layer film to a double-layer film, and the R_p_ value decreased [19]. When the potential was 1.75 V, the R_p_ value increased, because a dense single-layer oxide film composed of TiO_2_ was formed on the surface of the composites. In concentrated H_2_SO_4_, the polarization resistance of the Ti_3_SiC_2_/Cu composites increased with the increase in the potential. With the increase in the potential, the R_b_ value increased, because the doping ion made it thinner, with much larger surface area, causing the increase in the resistance of the positive current flow. As the potential increased, the R_t_ value decreased, because at the specific potentials the doping ions formed an energy barrier, which was related to the structural adjustment. On the other hand, it promoted the performance of the reverse current, decreasing the charge-transfer resistance [22,23,24]. 

### 3.3. Surface Morphology and Composition of Polarization Surface

**Figure 4:**
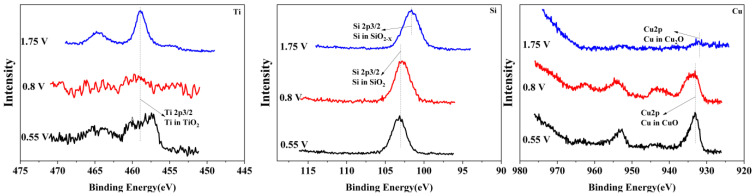
The XPS spectra of Ti, Si and Cu for Ti_3_SiC_2_/Cu soaking in dilute HNO_3_ (11.6%) at 0.55 V, 0.8 V and 1.75 V.

**Figure 5:**
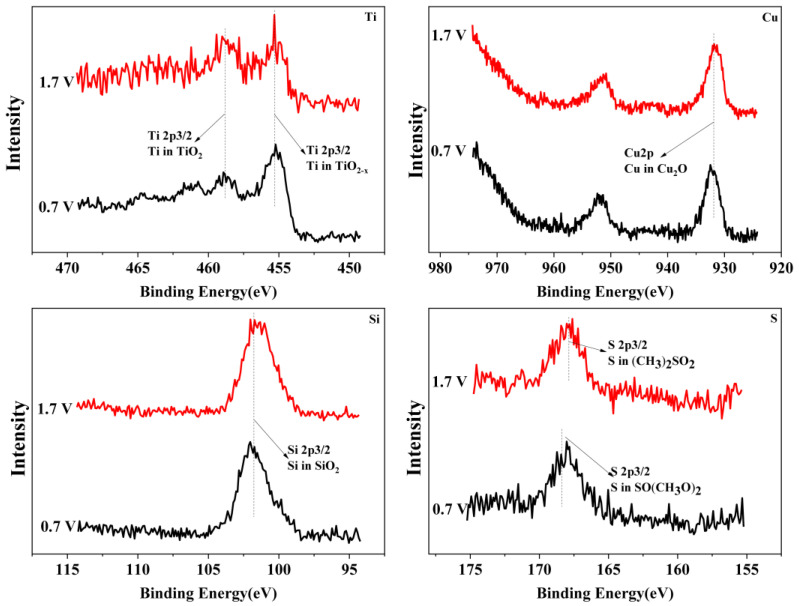
The XPS spectra of Ti, Cu, Si and S for Ti_3_SiC_2_/Cu potentiostatic polarization in concentrated H_2_SO_4_ at 0.7 V and 1.7 V.

To reveal the passivation mechanism of the Ti_3_SiC_2_/Cu composites, XPS (see Figure 4 and Figure 5) was applied to investigate the chemical composition of the passivation surface under various voltages. As shown in Figure 4 and Figure 5, the chemical composition of the composites in two strong acids were obviously different. In HNO_3_ (11.6%), under all potentials, TiO_2_ positioned at 464 eV and 459 eV (see Figure 4) was detected for Ti 2p. However, in concentrated H_2_SO_4_, the Ti 2p was examined in the shape of TiO_2_ or TiO_2−X_ (positioned at 455 eV and 459 eV). The Si 2p was measured in the form of SiO_2_ in dilute HNO_3_ (11.6%) (see Figure 4), while it was present as SiO_2_ or SiO_2-X_ (positioned at 103 eV and 101 eV) in concentrated H_2_SO_4_ (see Figure 5). The Cu 2p was detected in the shape of CuO or Cu_2_O (positioned at 933 eV and 931 eV) in dilute HNO_3_ (11.6%), while it was present as Cu_2_O in concentrated H_2_SO_4_. After treatment in concentrated H_2_SO_4_, S2p was seen on the surface of the composites in the shape of SO(CH_3_O)_2_ or (CH_3_)_2_SO_2_ (positioned at 168 eV and 169 eV).

The chemical composition of the composites after immersion in two strong acids were compared. In dilute HNO_3_ (11.6%), with the increase in the potential, Ti 2p was examined only as TiO_2_, while those of Si and Cu varied with the potential. When the potentials were 0.55 V and 0.8 V, the Si and Cu were present in the shapes of SiO_2_ and CuO, respectively. As the voltage increased, for example, 1.75 V, SiO_2_ and CuO were transformed into SiO_2−X_ and CuO_2−X_. In comparison, in concentrated H_2_SO_4_, with the increase in the potential, the Ti was always examined in the shapes of TiO_2_ and TiO_2−X_. Additionally, the Si and Cu were present as SiO_2_ and Cu_2_O, respectively. Interestingly, the S existed in different oxidation states with the change in the potential. Under low potentials (for example, 0.7 V), S 2p was present as SO(CH_3_O)_2_. When the potential increased, SO(CH_3_O)_2_ was transformed to (CH_3_)_2_SO_2_. 

### 3.4. Corrosion Mechanism

**Figure 6:**
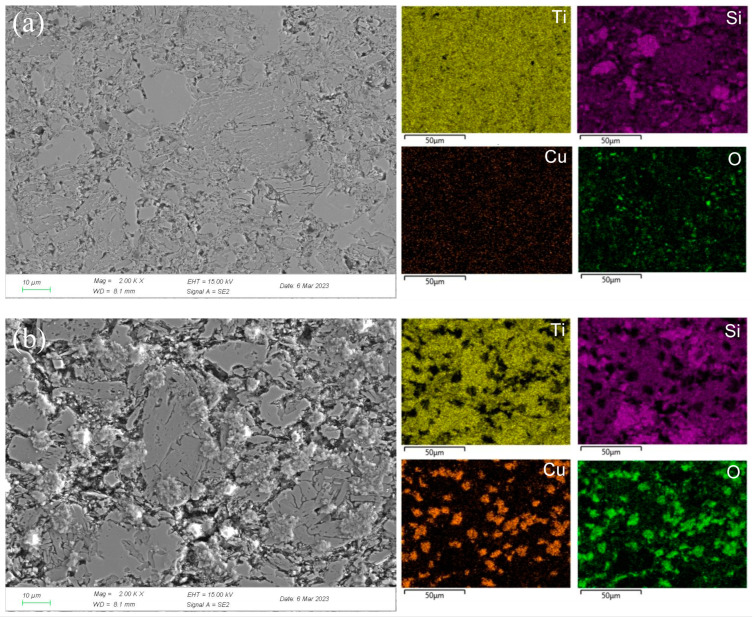
SEM micrographs and the corresponding elemental mapping distribution of Ti_3_SiC_2_/Cu after potentiostatic polarization in dilute HNO_3_ (11.6%) for 12 h at 0.55 V (**a**) and 0.8 V (**b**).

**Figure 7:**
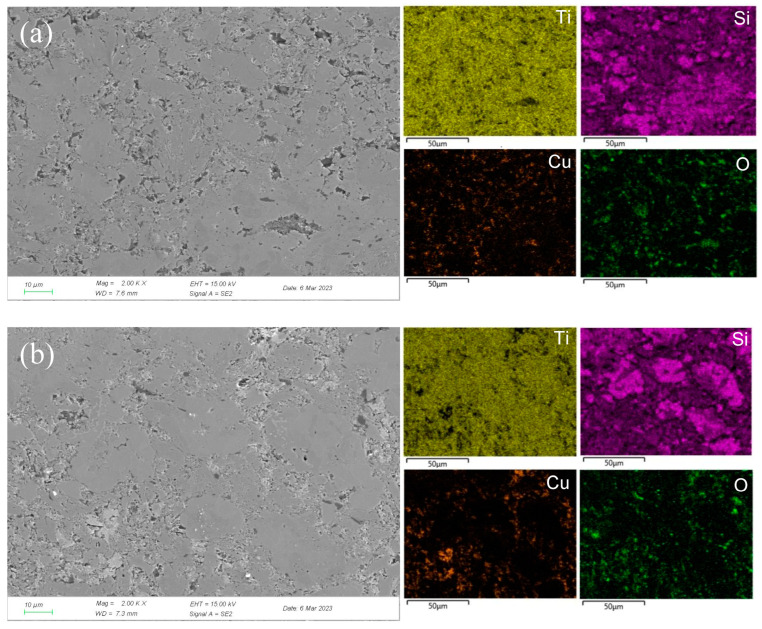
SEM micrographs and the corresponding elemental mapping distribution of Ti_3_SiC_2_/Cu after potentiostatic polarization in concentrated H_2_SO_4_ for 12 h at 0.7 V (**a**) and 1.7 V (**b**).

**Figure 8:**
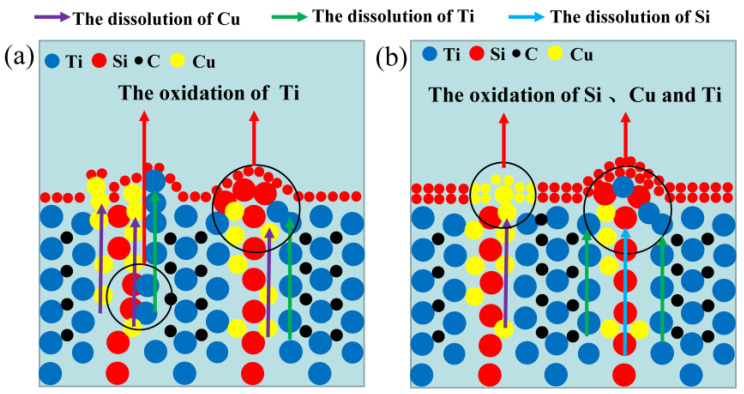
The passivation schematic of Ti_3_SiC_2_ in (**a**) dilute HNO_3_ (11.6%) and (**b**) concentrated H_2_SO_4_.

To clearly explore the electrochemical corrosion mechanism in harsh media, the corrosion morphology of the Ti_3_SiC_2_/Cu composites (see Figure 6 and Figure 7) was analyzed, accompanied with a proposed-mechanism diagram (see Figure 8). In dilute HNO_3_, the Ti-Si bonds and Cu-Si bonds among the composites were relatively weak. During the corrosion process, Cu atoms and Si atoms were first dissolved. Then, the Si atoms diffused to the outside and were oxidized. On the other hand, the Ti atoms were oxidized in situ. Therefore, a double-layer oxide film consisting of an internal layer of TiO_2_ and an external layer of SiO_2_ formed. When the potential (from OCP to 0.2 V) was relatively low, the main reaction was reaction (1). As the voltage was >0.2 V, the dominant reaction was reaction (2) and a single-layer oxide film was generated. The character of the layered structure of the composites and the weak interaction of Cu-Si and Ti-C was beneficial for the outside diffusing of Cu and Si atoms, accelerating the reaction (2). Thus, a large amount of Cu and Si atoms was lost (see Figure 8a). At 0.55 V, the passivation gap was not wide, because the dissolution of Cu-Si compounds was favorable for the formation of holes or defects, resulting in the destruction of the passivation layer (see Figure 6a). When the potential was 0.8 V, the current density decreased again, and a passivation interval appeared, too. It was guessed that Cu atoms were oxidized around the holes or defects, repairing the passivation layer and forming a double-layer oxide film comprising an internal layer of TiO_2_ and an external layer of Cu_2_O and SiO_2_. However, the CuO formed was dissolved by the highly corrosive HNO_3_. According to Figure 1, when the potential was 1.4 V, the current density began to decrease, because the outer layer of SiO_2_ was dissolved and the inner layer of TiO_2_ was exposed.

In concentrated H_2_SO_4_, Ti-Si and Cu-Si bonds were first broken, then Cu and Si atoms diffused into the outside and were oxidized into Cu_2_O and SiO_2_, respectively. Moreover, Ti atoms were oxidized in situ. A double-layer oxide film consisting of an internal layer of TiO_2_ and an external layer of Cu_2_O and SiO_2_ was formed (see Figure 8b). When the potential (from OCP to 0.1 V) was relatively low, the main reaction was reaction (1). As the voltage was >0.1 V, the reaction (2) became the dominant reaction and a passivation layer was formed. With the increase in the potential, Cu-Si was seriously destroyed, resulting in the failure of the passivation layer. As shown in Figure 7 and Table 3, at 0.7 V, a few corrosion pits were detected on the surface of the Ti_3_SiC_2_/Cu composites and the atomic percentage of Cu decreased, which resulted from the dissolution of the Cu-Si compounds. In the passivation range, the surface of the composites was covered by a double-layer oxide film comprised of TiO_2_, SiO_2_ and Cu_2_O. Compared to the electrochemical corrosion morphology of other metal matrix composites in sulfuric acid, the corrosion samples in this experiment are more complete [25,26]. With the increase in the potential, the dissolution of Cu atoms led to the destruction of the passivation layer. As the voltage was 1.7 V, there was a large amount of Cu_2_O around the corrosion pits. A peak around 1 V appeared, and the generation of Cu_2_O on the surface repaired the oxide film. Additionally, TiO_2−x_ was transformed into TiO_2_. Therefore, the corrosion resistance of the composites was improved. It was well known that it was possible for the oxide film of the Ti-containing material to transform from amorphous to crystalline under low potentials, causing the appearance of the second peak (see Figure 1). As the voltage was >1.4 V, a secondary passivation occurred for the Ti_3_SiC_2_/Cu composites. At this moment, TiO_2−x_ began to transformed into TiO_2_, forming a dense single-layer oxide film.

## 4. Conclusions

The electrochemical corrosion behaviors of the Ti_3_SiC_2_/Cu composites in dilute HNO_3_ and concentrated H_2_SO_4_ were studied. The results show that the Ti_3_SiC_2_/Cu composites have better electrochemical corrosion resistance in concentrated H_2_SO_4_. In dilute HNO_3_ (11.6%) solution, when the potential reaches 0.55 V the formation of a single layer of oxide film occurs; when the potential continues to rise, the Ti_3_SiC_2_/Cu surface tends to stabilize, the corrosion current density appears to be substantially decreased, and the material on the surface of the oxide film gradually becomes dense. In the concentrated H_2_SO_4_ solution, when the potential reaches 0.7 V, the Ti_3_SiC_2_/Cu composite surface formed a double-layer oxide film, thus improving the corrosion resistance. With the increase in potential, the oxide film is more stable and the corrosion resistance is stronger.

## Figures and Tables

**Table 1:** The electrochemical parameters of Ti_3_SiC_2_ and Ti_3_SiC_2_/Cu in dilute HNO_3_ and concentrated H_2_SO_4_.

	E_corr_ (V)	i_corr_ (A/cm^2^)	i_p_ (A/cm^2^)	E_p_ (V)
HNO_3_ (11.6%)	0.06	7.05 × 10^−4^	5.01 × 10^−5^	0.55
H_2_SO_4_	0	3.01 × 10^−6^	6.87 × 10^−6^	0.6

**Table 2:** The equivalent circuit fitting parameters of Ti_3_SiC_2_/Cu in dilute HNO_3_ and concentrated H_2_SO_4_ under different conditions.

		R_s_ (Ω·cm^2^)	Q_1_ (10^−4^F/cm^2^)	R_t_ (Ω·cm^2^)	Q_2_ (10^−4^ F/cm^2^)	R_b_ (Ω·cm^2^)
HNO_3_ (11.6%)	OCP	0.7151	120.38	9.785	118,600	4.79
	0.55 V	0.84	609.96	14.66	816.66	34.75
	0.8 V	0.7277	127.52	9.778	17,630	8.679
	1.75 V	1.023	143.88	7.129	2875.1	17.46
H_2_SO_4_	OCP	5.67	8.91	430.9	24.192	1843
	0.7 V	5.251	6.81	300.1	10.417	6731
	1.7 V	4.744	1.52	77.81	13	15,275

**Table 3:** Atomic ratios of Ti_3_SiC_2_/Cu in dilute HNO_3_ and concentrated H_2_SO_4_.

Samples		Atomic Percentages
HNO_3_ (11.6%)	0.55 V	37.3%Ti, 11.17%Si, 31.28%C, 0.66%Cu, 17.75%O
	0.8 V	22.48%Ti, 11.01%Si, 18.82%C, 4.25%Cu, 41.54%O
	1.75 V	38.2%Ti, 13.22%Si, 27.17%C, 0.34%Cu, 19.06%O
H_2_SO_4_	0.7 V	34.59%Ti, 17.72%Si, 29.82%C, 2.91%Cu, 17.72%O, 0.13%S
	1.7 V	34.25%Ti, 13.94%Si, 31.26%C, 5.45%Cu, 13.94%O, 0.06%S

## Data Availability

Data are contained within the article.
